# The Use of a Hemi Glabellar Flap for Reconstruction of Medial Canthus Defects

**DOI:** 10.7759/cureus.21880

**Published:** 2022-02-03

**Authors:** Poh Hong Tan, Khemerin Eng, Joshua Agilinko, Amr S Khalil

**Affiliations:** 1 Plastic Surgery, Wythenshawe Hospital, Manchester, GBR; 2 Surgery, St James's University Hospital, Leeds, GBR; 3 General Surgery, Whipps Cross, London, GBR; 4 Plastic Surgery, Transform Hospital Group, Pines Hospital, Manchester, GBR

**Keywords:** glabellar flap, hemi glabellar flap, medial canthus defect, facial defect, skin repair

## Abstract

Background: Medial canthal reconstruction is a challenging task due to its complex anatomy. The glabellar flap is a common viable technique; however, this results in narrowing of the eyebrows, bulky nasal dorsum horizontal scarring, which is aesthetically displeasing, and possible injury of the supratrochlear artery. Multiple variations have been proposed in the literature, which is often complex. In this paper, the senior author (AK) has developed an intuitive, simple technique by utilising half of the glabellar skin in 12 patients with good clinical outcomes.

Materials and methods: A rotational advancement flap involving the upper lateral nasal wall with the hemi glabellar was formed and transferred to the medial canthal defect. The donor site was closed in a V-Y manner. Complete closure of defect was achieved in all patients.

Results: Reconstruction using the hemi glabellar technique was performed on 12 patients following resection of basal cell carcinoma (BCC) in or near the medial canthus area. Superficial cellulitis was noted in two patients; they were managed on oral antibiotics. Bruising was reported in seven patients which resolved spontaneously in 4-7 days. All patients had a good outcome at two months and six months follow up; there was no flap loss, and all patients were satisfied with the aesthetic outcome.

Conclusion: The technique highlighted in this article can be performed quickly and applies to the reconstruction of medial canthus defects with excellent aesthetic outcomes, an inconspicuous scar and supple skin with matching colour.

## Introduction

Reconstruction of a medial canthus defect is challenging due to the complex anatomical structure of the lateral nasal wall and the upper and lower eyelid junction [1]. It is important to avoid distortion of the canthus concavity and maintain eyebrow contour and symmetry during reconstruction.

Several methods have been described, including healing by secondary intention, full or partial thickness skin grafts, and local flaps [2-7]. One of the favored techniques is the glabellar flap because the color, texture, and thickness match with inconspicuous scars.

The glabellar flap is a rotation advancement flap of the glabella to cover wounds up to 2.5 cm. Its blood supply is often described as random axial, but it can be based on the supratrochlear or angular artery in some cases [7].

The main advantages of the classical glabellar flap technique in periocular reconstruction include less contracture than a skin graft, a good blood supply due to its broad base, minimal local structural damage, and overall good aesthetic result [5]. The specific disadvantages are narrowing of the eyebrows when the V is closed to become a Y, which can require more procedures such as laser removal of eyebrow hair, a bulky nasal dorsum due to the skin and subcutaneous tissue of the glabella, horizontal scarring, and possible injury of the supratrochlear artery [1].

Several modifications of the classical glabellar flap have been proposed, such as forming a redundant triangular tip [2], combined glabellar and rintala flap [7], and the “flap in flap” technique [5] with variable clinical outcomes. However, these techniques are often complex and can require a two-stage procedure.

This article presents a simple, intuitive modification of the glabellar rotation advancement flap that is easy to adopt and produces a better aesthetic outcome. We have named this modification “the hemi glabellar flap.”

## Technical report

Method

The hemi glabellar flap was used by the senior author (AK) to reconstruct 12 medial canthal defects after basal cell carcinoma (BCC) excision between 2017 and 2020. BCC excision was performed according to British Association of Dermatology guidelines [8]. All operations were performed as a one-stage procedure under local anesthesia.

The Hemi Glabellar Flap Technique

Following preparation and surgical draping of the site, an outline of the flap was marked by drawing an inverted V involving the medial or lateral half of the glabella on the defect side. The area was then anesthetized using 2% lidocaine with adrenaline 1:80000.

An incision is made with a #15 scalpel blade deep to the glabella crease. The plane starts at the subcutaneous layer of the glabella and reaches the submuscular plane in the lateral nasal wall. Care is required to preserve the eyebrow depressor muscle. The flap is advanced then rotated to its final location. The defect closed in a V-Y manner. The flap is then trimmed to fit the medial canthus defect. After adequate hemostasis, both wounds are closed in two layers: a deep layer with 5-0 Monocryl® (Ethicon Inc., Somerville, NJ) or monofilament and 6-0 nylon for the skin. Steri-strips are then used for extra dressing.

Further thinning of the flap can be done without compromising the skin due to the vast network of blood vessels.

Case study

Patient One

**Figure 1:**
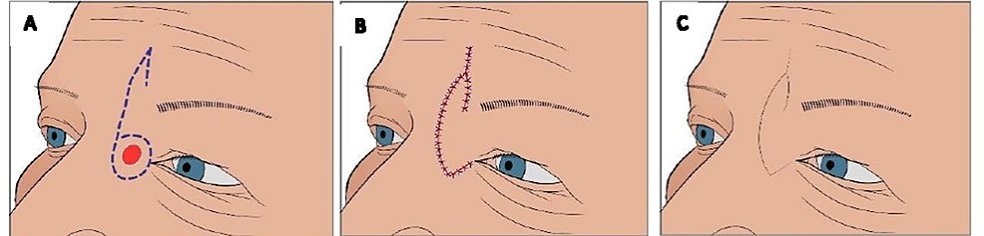
Glabella rotation flap over left medial canthus. (A) preop marking of lesion and flap. (B) excision of lesion and flap rotated inferiorly into defect. (C) patient at eight week follow-up.

A 46-year-old woman was evaluated for a nodular, beaded, non-ulcerating lesion with well-defined borders on the lateral nasal sidewall near the medial canthus. She had had the lesion for nine months, and a general physician initially diagnosed it as a cyst. She was referred to the plastic surgery clinic due to a non-healing nodule. A nodular BCC was diagnosed, and the lesion was resected with a 4-5 mm margin with immediate reconstruction. A lateral-based hemi glabella flap was created to cover the 2.3 cm defect. Sutures were removed after a week, and the wound healed well. Clear margins were described in the histopathology report. The patient had an excellent cosmetic outcome at six months follow-up and was satisfied (Figure 1).

Patient Two

A 52-year-old woman was evaluated for a slow-growing bleeding ulcerated nodule in the right medial canthus of four months evolution. The lesion was 1.5 cm in width with beaded edges and well-defined borders. Otherwise, she was fit and well.

**Figure 2:**
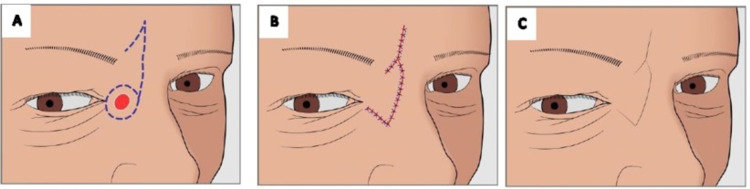
Glabella rotation flap over right medial canthus. (A) preop marking of lesion and flap. (B) excision of lesion and flap rotated inferiorly into defect. (C) patient at eight week follow-up.

The lesion was excised with a 4-mm margin involving the medial canthus ligament. Primary reconstruction was carried out using a hemi glabellar flap. This procedure was done as a day case under local anesthesia. The wound was closed with a 4.0 Monocryl® subcutaneous suture and 5.0 Ethilon® (Ethicon, New Brunswick, NJ) in the skin. The patient was seen at the dressing clinic one week after surgery. The sutures were removed, and the flap was viable. At week eight after surgery, the wound was slightly erythematous but healing well. The pathological description reported clear margins. At six months, the scar was inconspicuous, and the patient was satisfied (Figure 2).

Results

The senior author has performed the hemi glabellar flap on ten women and two men between 45 and 65. All defects were from BCC excisions. The excised tumor had clear margins except in one patient. In this patient, a decision to “watch and wait” was made by a multidisciplinary team. There was no recurrence at six months follow-up, and the patient was discharged. All patients underwent BCC excision with immediate reconstruction of the medial canthus since the BCC had clinically well-defined borders.

Sutures were removed in 7-10 days with excellent results in all patients. The wound had completely healed at eight weeks of follow-up, and at six months, there was minimal scarring. All 12 patients were happy with the aesthetic outcome and were discharged.

Bruising around the forehead and eyelid was seen in 60% of the patients but this was resolved without intervention after 4-7 days. There was pin cushioning deformity in two patients; however, the patients were satisfied and did not request further intervention. Two patients developed superficial cellulitis that responded well to antibiotics. There was no flap loss or necrosis in this case series.

## Discussion

The medial canthus is a highly complex region that involves various skin textures, thicknesses, and contours. These include the medial eyebrow, the lateral skin of the dorsum of the nose, the upper cheek, and the eyelid. For example, the skin of the medial canthus is thicker than the skin on the eyelid but thinner than the skin of the nasal dorsum and the skin of the eyebrows. Moreover, important structures deep in the skin, such as the canthal ligaments and the lachrymal passage system [4], make reconstruction difficult.

Some surgeons prefer secondary intention healing; however, this method may take 4-6 weeks to heal and will result in a significant scar that is often unacceptable to patients. A full-thickness or partial-thickness skin graft is an alternative method; however, a depressed area with a color mismatch and a scaly texture is produced, especially in a split skin graft. In addition, this is aesthetically unacceptable to patients.

The classical glabellar flap is a viable reconstruction technique to overcome these disadvantages. It closely matches color and texture and has its blood supply. It is also cosmetically superior to the techniques mentioned above. Various modifications of the glabellar flap exist depending on the size of the defect and counter the common disadvantages stated before.

Turgut et al. [5] described a 'flap in flap' technique that divides the glabella into two smaller segments via a diagonal line. The closer limb is then transposed into the defect while the larger limb covers the donor site. Meadows et al. [2] salvaged the glabellar flap tip to fill the secondary defect rather than primary closure. While both articles report the prevention of interbrow distance narrowing, there is a high risk of flap necrosis due to the nature of the flap.

Chao et al. [9] proposed complex techniques involving the rintala flap. While these procedures effectively reduced the bulkiness in the nasal dorsum and prevented narrowing of the interbrow distance, both techniques were difficult to achieve with more potential complications.

The technique highlighted in our case series adequately achieves medial canthus reconstruction without risking vascular and structural components of the flap and nasal dorsum and maintaining a natural interbrow distance.

The critical advantage here is the ease of use and overall efficacy compared to other modifications in the literature. The main disadvantage is the potential injury to the supratrochlear artery, pin cushioning, and the thickness of the flap.

## Conclusions

In summary, we found a simple and easy modification to the glabellar flap technique, which is robust and addresses the common shortcomings of the traditional technique and current methods in medial canthus reconstruction. In this article, the senior author has successfully performed 12 procedures with the technique discussed. All 12 patients had excellent aesthetic outcomes at the eight weeks and six months follow up with minimal complications. This outcome was comparable to other techniques in the literature but is significantly easier to perform. Therefore, this article recommends this modified glabellar flap technique for medial canthus reconstruction following excision of skin cancer. 
